# Hypercapnia Alters Expression of Immune Response, Nucleosome Assembly and Lipid Metabolism Genes in Differentiated Human Bronchial Epithelial Cells

**DOI:** 10.1038/s41598-018-32008-x

**Published:** 2018-09-10

**Authors:** S. Marina Casalino-Matsuda, Naizhen Wang, Peder T. Ruhoff, Hiroaki Matsuda, Marie C. Nlend, Aisha Nair, Igal Szleifer, Greg J. Beitel, Jacob I. Sznajder, Peter H. S. Sporn

**Affiliations:** 1 Department of Medicine, Feinberg School of Medicine, Northwestern University, Chicago, Illinois United States of America; 2 Department of Technology and Innovation, University of Southern Denmark, Odense, Denmark; 3 Department of Biomedical Engineering, Northwestern University, Evanston, Illinois United States of America; 4 Department of Physical Sciences & Engineering, Wilbur Wright College, Chicago, Illinois United States of America; 5 Division of Protein and Cellular Analysis, Thermo Fisher Scientific, Rockford, Illinois United States of America; 6 Department of Chemistry, Northwestern University, Evanston, Illinois United States of America; 7 Chemistry of Life Processes Institute, Northwestern University, Evanston, Illinois United States of America; 8 Department of Molecular Biosciences, Northwestern University, Evanston, Illinois United States of America; 9 Jesse Brown VA Medical Center, Chicago, Illinois United States of America

## Abstract

Hypercapnia, the elevation of CO_2_ in blood and tissues, commonly occurs in severe acute and chronic respiratory diseases, and is associated with increased risk of mortality. Recent studies have shown that hypercapnia adversely affects innate immunity, host defense, lung edema clearance and cell proliferation. Airway epithelial dysfunction is a feature of advanced lung disease, but the effect of hypercapnia on airway epithelium is unknown. Thus, in the current study we examined the effect of normoxic hypercapnia (20% CO_2_ for 24 h) vs normocapnia (5% CO_2_), on global gene expression in differentiated normal human airway epithelial cells. Gene expression was assessed on Affymetrix microarrays, and subjected to gene ontology analysis for biological process and cluster-network representation. We found that hypercapnia downregulated the expression of 183 genes and upregulated 126. Among these, major gene clusters linked to immune responses and nucleosome assembly were largely downregulated, while lipid metabolism genes were largely upregulated. The overwhelming majority of these genes were not previously known to be regulated by CO_2_. These changes in gene expression indicate the potential for hypercapnia to impact bronchial epithelial cell function in ways that may contribute to poor clinical outcomes in patients with severe acute or advanced chronic lung diseases.

## Introduction

Hypercapnia, the elevation of CO_2_ in blood and tissue, commonly occurs in patients with acute respiratory failure and chronic pulmonary disorders^1–3^. It has long been recognized that hypercapnia is associated with an increased risk of death in chronic obstructive pulmonary disease (COPD)^4–8^. Other studies have shown that hypercapnia is an independent risk factor for mortality in adults with community-acquired pneumonia^9^, children with adenoviral pneumonia^10^, cystic fibrosis patients awaiting lung transplantation^11^, and patients receiving mechanical ventilation for acute respiratory distress syndrome (ARDS)^12^.

Recent research has shed light on a variety of mechanisms by which hypercapnia may adversely affect clinical outcomes in patients with lung disease. We and others have shown that elevated CO_2_ inhibits LPS-induced expression of IL-6 and TNF in macrophages, fibroblasts and alveolar epithelial cells^13,14^. We also found that hypercapnia impairs bacterial clearance through inhibition of both phagocytosis and autophagy in macrophages^13,15^. Moreover, hypercapnia worsened lung injury and decreased bacterial clearance in mechanically ventilated rats with *E. coli* pneumonia^16^ and increased the mortality of *Pseudomonas* pneumonia in mice^17^. In the latter study, elevated CO_2_ decreased the early release of TNF, IL-6 and multiple chemokines into the lung, inhibited bacterial phagocytosis and NADPH-oxidase-mediated reactive oxygen species generation by lung neutrophils, and increased bacterial loads in the lungs and other organs^17^. Hypercapnia was also shown to inhibit alveolar fluid clearance in the rat lung, which was due to downregulation of Na,K-ATPase activity caused by endocytosis of the enzyme due to activation of AMP-activated kinase and PKC-zeta^18,19^. Additionally, elevated CO_2_ inhibited proliferation of alveolar epithelial cells and lung fibroblasts, which resulted from mitochondrial dysfunction triggered by mirR-183-dependent downregulation of isocitrate mitochondrial dehydrogenase 2 (IDH2)^20^. Thus, hypercapnia adversely affects innate immune responses, host defense, lung edema clearance, and proliferation of cells required for lung repair. Notably, in almost all of the foregoing studies, elevated CO_2_ produced these effects independently of acidosis.

While the above studies have focused on the impact of hypercapnia on macrophages, neutrophils, fibroblasts, and alveolar epithelial cells, how elevated CO_2_ affects bronchial epithelial cells has not previously been investigated. The airway epithelium is the first line of defense against inhaled pathogens and other noxious agents, and its integrity is critical for host defense and maintenance of lung homeostasis^21,22^. Also, diseases associated with hypercapnia including COPD, asthma, cystic fibrosis and ARDS are all characterized by airway epithelial dysfunction^23–26^. Thus, in the current study we examined the effect of hypercapnia on global gene expression in normal human bronchial epithelial (NHBE) cells that were differentiated at the air-liquid interface (ALI). We show that exposure for 24 h to normoxic hypercapnia (20% CO_2_), as opposed to normocapnia (5% CO_2_) downregulates genes linked to innate immunity, host defense, and nucleosome assembly and upregulates genes required for cholesterol biosynthesis and lipid metabolism. These changes in gene expression indicate the potential for hypercapnia to alter bronchial epithelial cell function in ways that may contribute to poor clinical outcomes in patients with severe acute or advanced chronic lung diseases.

## Results

### Differential gene expression induced by hypercapnia

**Figure 1:**
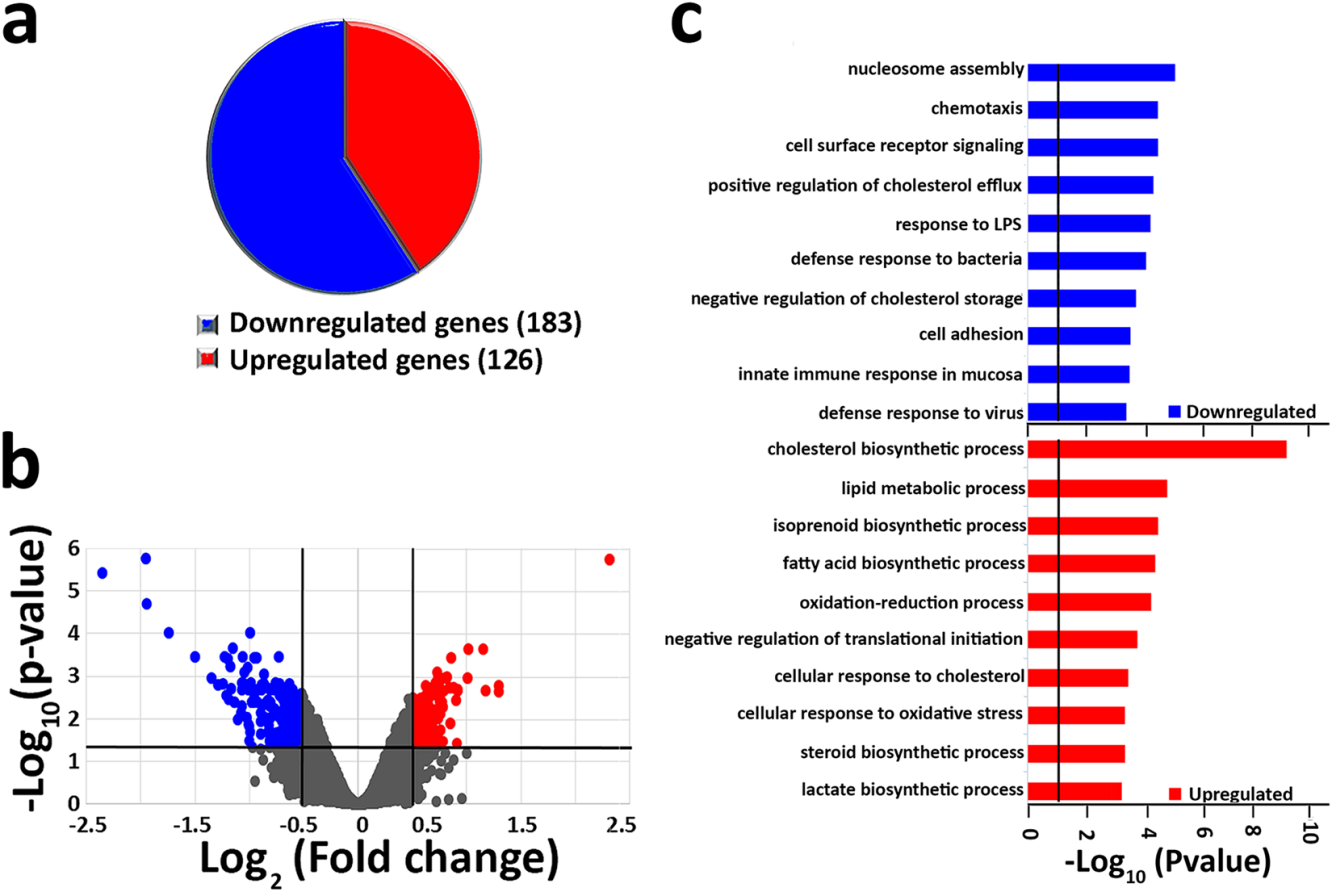
Hypercapnia induces transcriptional changes in NHBE cells. Global gene expression was assessed in ALI-differentiated NHBE cells after exposure to 5% CO_2_ (normocapnia) or 20% CO_2_ (hypercapnia) for 24 h. (**a**) Pie chart indicating proportion of genes downregulated or upregulated by hypercapnia. (**b**) Volcano plot showing statistical significance (−log_10_ [P value]) plotted against log_2_ fold change for hypercapnia vs normocapnia. Plot indicates significantly upregulated genes (log_2_ [fold change]≥+0.5, adjusted P value ≤ 0.05) in red and downregulated genes (log_2_ [fold change]≤−0.5, adjusted P value ≤ 0.05) in blue. (**c**) Bars represent the top 10 GO biological processes downregulated (blue) and upregulated (red) by high CO_2_.

To determine whether elevated CO_2_ induces transcriptional changes in airway epithelium, NHBE cells differentiated at ALI were exposed to hypercapnia (20% CO_2_) for 24 h or maintained in normocapnia (5% CO_2_) as a control, and global gene expression was analyzed using Affymetrix GeneChip Hybridization. Microarray global gene expression analysis revealed that hypercapnia significantly modified the expression of 309 genes ≥±1.4 fold (expressed as log_2_ [fold change]) with an adjusted P value ≤ 0.05. This represents only 1.5% of the 20,390 transcripts represented on the Affymetrix chip, indicating that the impact of elevated CO_2_ on gene expression is highly selective. The proportion of genes whose expression was significantly upregulated (126) or downregulated (183) in response to high CO_2_ is depicted in Fig. 1a. Differential gene expression is also represented as a volcano plot of log_2_ (fold change ratio) vs. −log_10_ (P values) (Fig. 1b) and as a heat map with hierarchical clustering (Supplementary Fig. 1a). The names and fold-changes for all genes downregulated or upregulated by ≥1.4 fold are listed in Supplementary Tables 1,2, respectively. These transcriptional changes cannot be attributed to cytotoxicity since exposure to 20% CO_2_ for 24 h caused no increase in LDH release from the cells (Supplementary Fig. 2).

### Biological processes targeted by hypercapnia

Gene Ontology (GO) analysis showed that for hypercapnia-downregulated genes, the most enriched process was nucleosome assembly (Fig. 1c and Supplementary Fig. 1b), which includes multiple histone genes (Supplementary Fig. 1b). Other hypercapnia-downregulated processes include chemotaxis, cell surface receptor signaling, response to LPS, defense responses to bacteria, and cell adhesion (Fig. 1c). Specific CO_2_-downregulated genes associated with these processes are listed in Supplementary Fig. 1b. Among hypercapnia-upregulated genes, the most enriched processes involved lipid metabolism including cholesterol, isoprenoid, fatty acid and steroid biosynthesis, as well as oxidation-reduction, and negative regulation of translation initiation (Fig. 1c). Specific CO_2_-upregulated genes associated with some of these processes are listed in Supplementary Fig. 1b.

### GO biological process-associated gene clusters targeted by hypercapnia

**Figure 2:**
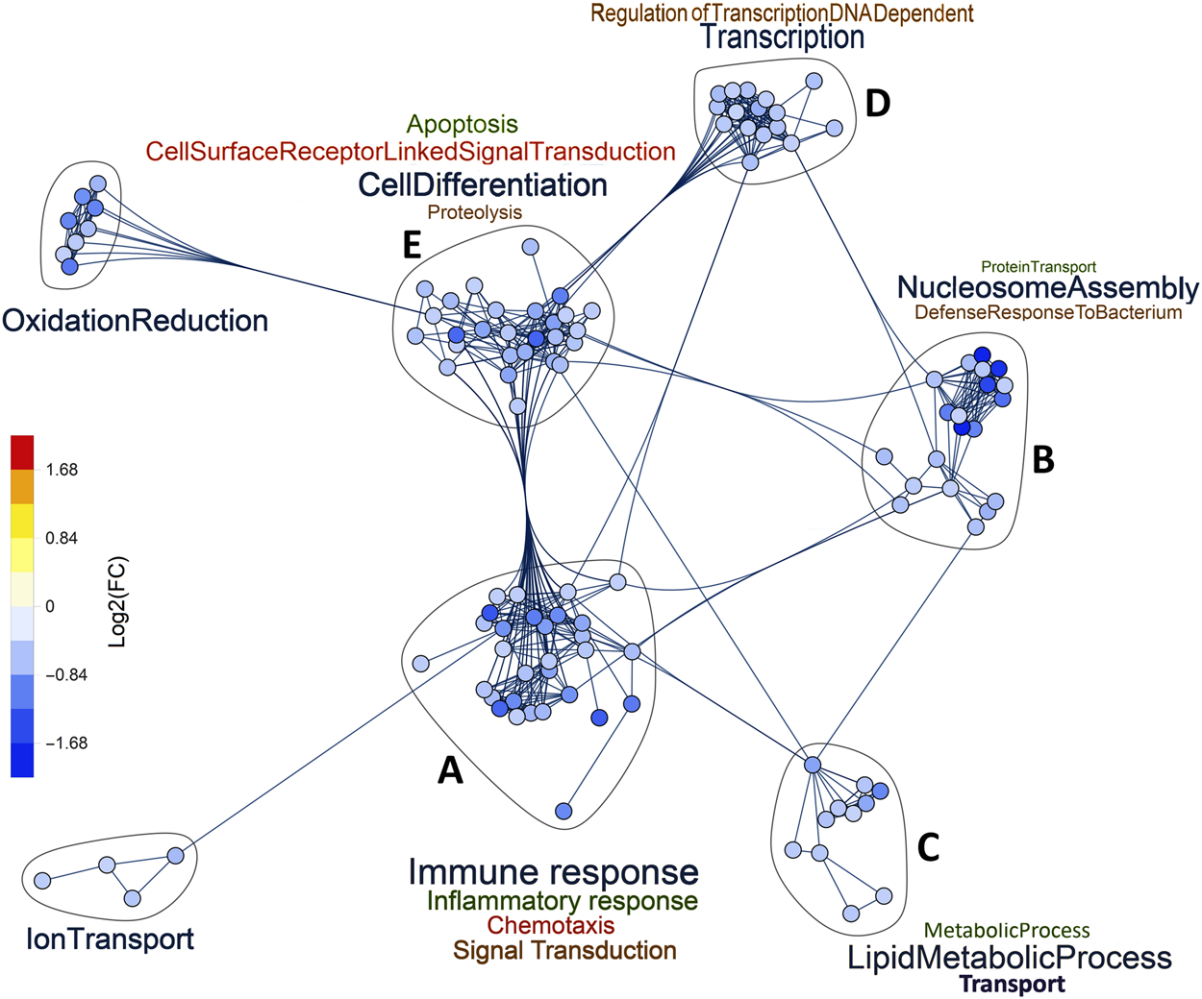
Networks of GO biological processes downregulated by hypercapnia. Gene clusters associated with GO biological processes containing five or more hypercapnia-downregulated genes and their intra- and inter-cluster connections, as determined by unbiased analysis using Mathematica® v11.2.

**Figure 3:**
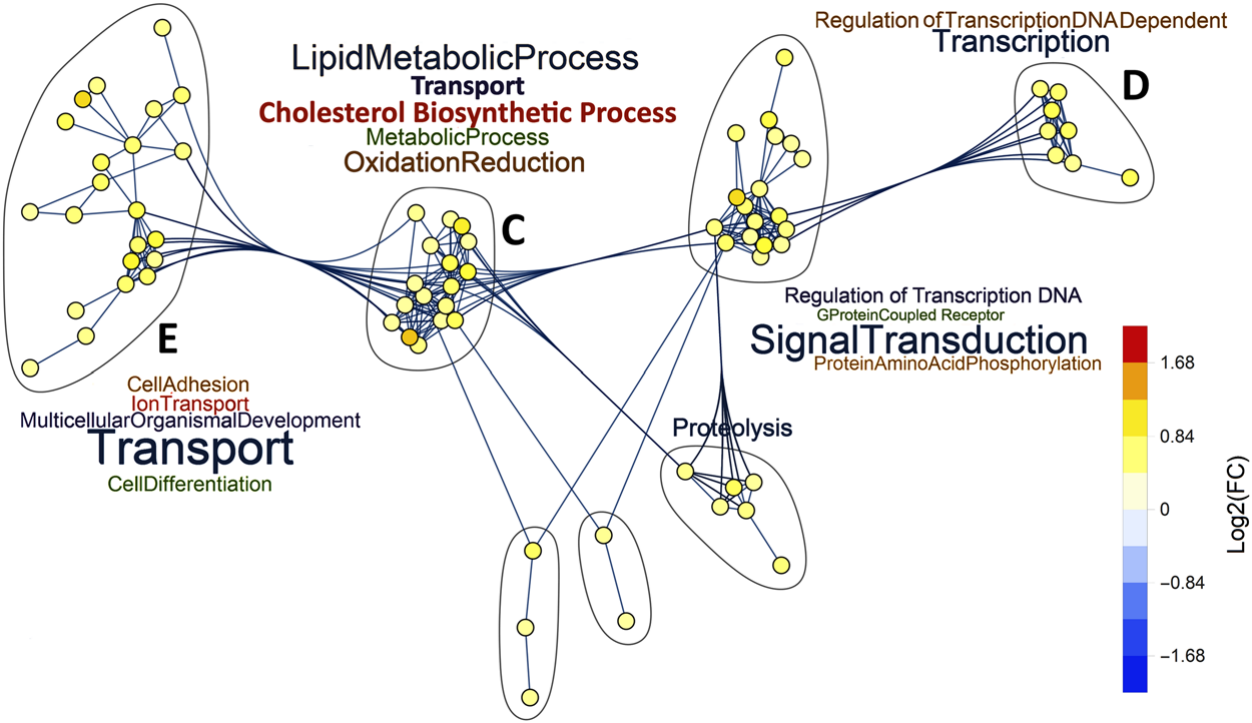
Networks of GO biological processes upregulated by hypercapnia. Gene clusters associated with GO biological processes containing five or more hypercapnia-upregulated genes and their intra- and inter-cluster connections, as determined by unbiased analysis using Mathematica® v11.2.

**Figure 4:**
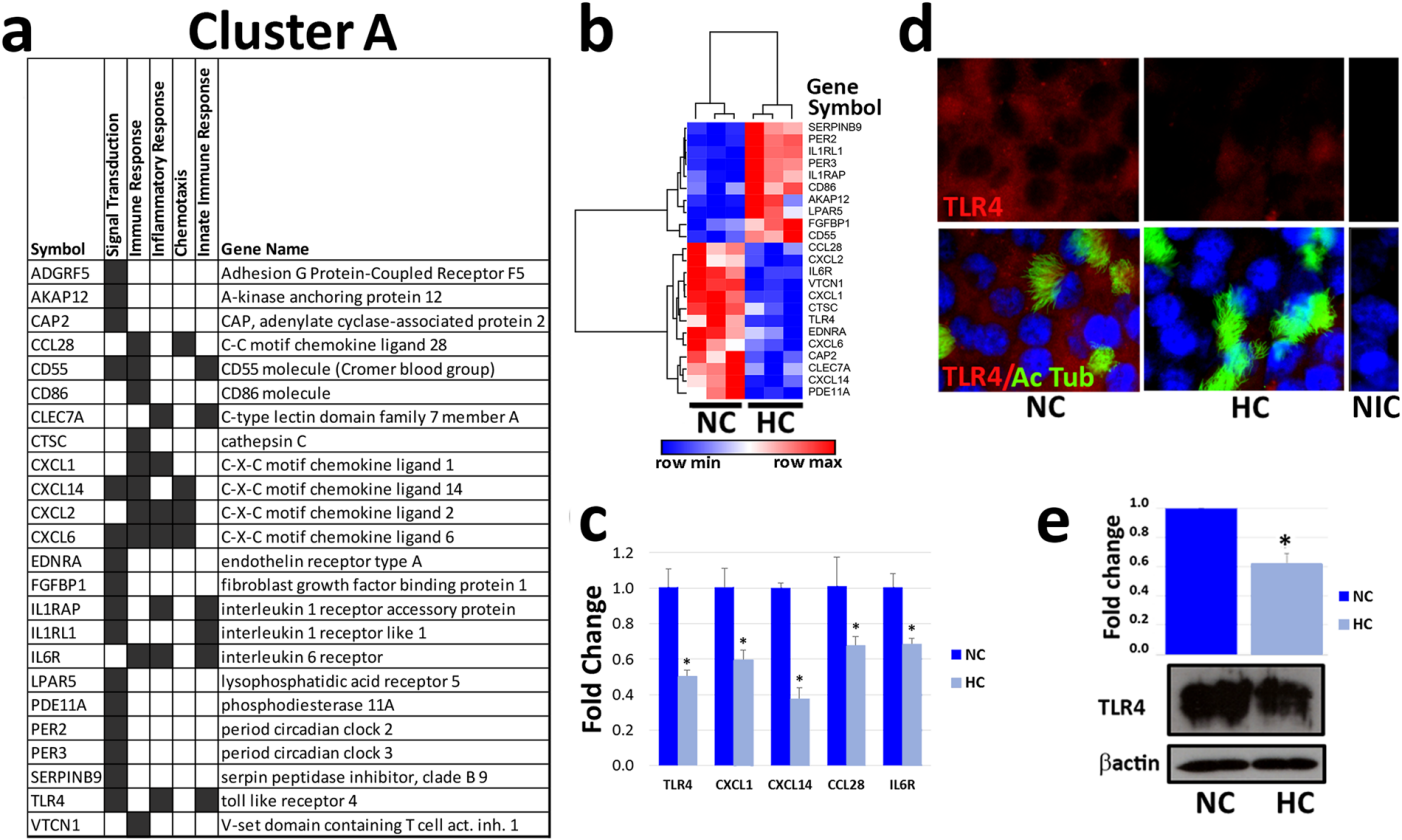
Hypercapnia alters expression of genes involved in innate immunity and host defense. ALI-differentiated NHBE cells were exposed to normocapnia (NC) or hypercapnia (HC) for 24 h prior to analysis. (**a**) Cluster A genes altered by hypercapnia and their associated GO biological processes. (**b**) Heatmap and hierarchical clustering of gene expression profiles in normocapnia and hypercapnia. (**c**) *CXCL1, CXCL14, CCL28, IL6R*, and *TLR4* mRNA expression levels were assessed by qPCR and expression in hypercapnia was expressed as fold change relative to normocapnia. Results shown are means ± SE; n = 3. (**d**) Representative fluorescence micrographs of NHBE cells double-stained for TLR4 (red) and the cilia marker acetylated tubulin (green), and counter stained with Hoechst (blue). Non immune control (NIC) cells stained without primary antibodies. (**e**) Immunoblotting of whole cell lysates for TLR4. Histogram shows densitometry of TLR4 normalized to β-actin (loading control). Results shown are means ± SE; n = 3.

**Figure 5:**
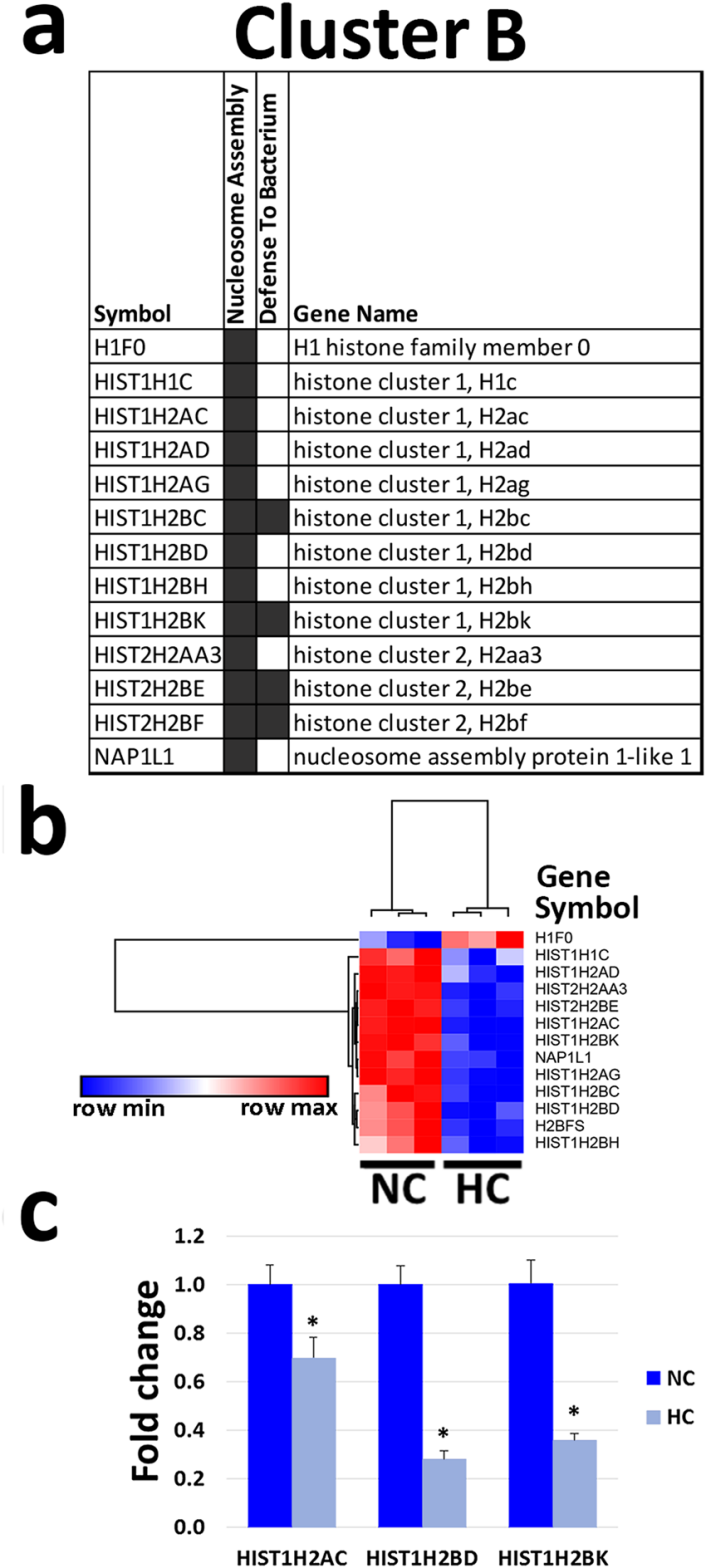
Hypercapnia alters expression of genes involved in nucleosome assembly. ALI-differentiated NHBE cells were exposed to normocapnia (NC) or hypercapnia (HC) for 24 h prior to analysis. (**a**) Cluster B genes altered by hypercapnia and their associated GO biologic processes. (**b**) Hierarchical clustering of the gene expression profiles in normocapnia and hypercapnia. (**c**) *HIST1H2AC, HIST1H2BD* and *HIST1H2BK* mRNA expression assessed by qPCR and hypercapnia was expressed as fold change relative to normocapnia. Results shown are means ± SE; n = 3.

Major clusters from hypercapnia-downregulated genes are linked to immune response, nucleosome assembly, cell differentiation, oxidation reduction, and ion and lipid transport (Fig. 2). Clusters from upregulated genes induced by high CO_2_ (Fig. 3) involve biological processes related to lipid metabolism, cholesterol biosynthesis, signal transduction, and transport. A number of these important clusters, labelled A-E in Figs 2 and 3, are analyzed in more detail in the following sections. Their corresponding gene lists are depicted in Figs 4,5, and Supplementary Fig. 3.

### Hypercapnia differentially regulates genes associated with innate immunity and nucleosome assembly

Cluster A, represented in Fig. 4a, includes hypercapnia-regulated genes involved in signal transduction, immune and inflammatory responses, and leukocyte chemotaxis. Notably, *TLR4*, multiple chemokines (*CCL28, CXCL1, CXCL2, CXCL6*, and *CXCL14*) and the IL-6 receptor gene (*IL6R*) were all downregulated by elevated CO_2_. On the other hand, the IL-1 receptor like 1 gene (*IL1RL1*) was upregulated by hypercapnia. To validate the microarray results related to CO_2_-induced changes in key immunoregulatory genes, expression of *CXCL1, CXCL14, CCL28, ILR6* and *TLR4* was also assessed by qPCR. We found that these genes were all downregulated at levels similar to those in the microarray analysis (Fig. 4c). Indeed, the degree of CO_2_-induced downregulation of these transcripts assessed by qPCR and microarray was highly correlated (r^2^ = 0.7981).

In addition, to determine whether downregulation of a key immunoregulatory transcript by hypercapnia was accompanied by a similar change in protein expression, we assessed expression of TLR4 protein in differentiated NHBE cells. Immunofluorescence microscopy (Fig. 4d) and immunoblotting (Fig. 4e) both showed that exposure to 20% CO_2_ for 24 h decreased NHBE cell expression of TLR4 protein. Full-length blots are included in Supplementary Fig. 4. Taken together, these results suggest that hypercapnia would suppress airway epithelial innate immune response to microbial pathogens and other inflammatory stimuli.

Next, we analyzed cluster B, which includes hypercapnia-regulated genes that codify proteins involved in nucleosome assembly (Fig. 5a). The heat map in Fig. 5b shows that hypercapnia downregulates genes encoding multiple family members of the core histones H2A and H2B^27^, as well as the nucleosome assembly protein 1-like 1 (*NAP1L1*), which regulates protein complex assembly, chromosome organization and DNA metabolism. The only upregulated gene in cluster B is *H1F0*, encoding histone H1, which is normally expressed in terminally differentiated and slowly dividing cells. To validate the microarray data from cluster B, we performed qPCR for selected transcripts whose expression was significantly altered in the microarray analysis. Figure 5c shows that expression of the histone genes *HIST1H2AC*, *HIST1H2BD*, and *HIST1H2BK* was downregulated by hypercapnia as assessed by qPCR, again similar to the microarray results.

### Other gene clusters impacted by hypercapnia

Cluster C (Supplementary Fig. 3) includes CO_2_-regulated genes associated with cholesterol biosynthesis (*DHCR7, FDFT1, HMGCS1, IDI1)* and transport (*ABCA1, ABCC3, ABCC5*), other lipid metabolism (*ALDH3B1, ALDH3B2, ALOX15B, FADS1–2*), and oxidation reduction (several members of the cytochrome P450 family). Cluster D (Supplementary Fig. 3) includes hypercapnia-regulated genes involved in transcription (*EGR3, JUN, RBM14*) and DNA repair (*BCCIP, CUL4A, RMB14*). Genes in cluster E (Supplementary Fig. 3) regulate cell differentiation (*CADM1, NRP2, NOTCH2NL* and others), cell surface receptor signaling (*EGFR, IFNAR1, IL6R* and others) and apoptosis (*BCL2L15, DAPL1, SEMA6A* and others). The impact of elevated CO_2_ on expression of these genes would be expected to alter epithelial metabolism and barrier function, as well as innate immune and inflammatory responses.

## Discussion

To our knowledge, the present study is the first to investigate the impact of hypercapnia on global gene expression in airway epithelial cells. Of importance, we utilized primary NHBE cells cultured at ALI to achieve a differentiated state closely resembling normal human bronchial epithelium. Our principal finding was that hypercapnia altered expression of a small number of specific genes (309 out of 20,390 transcripts assayed, or 1.5%) in differentiated NHBE cells. Of these, 183 genes (59%) were downregulated, while 126 (41%) were upregulated. Thus, the effects of elevated CO_2_ are highly selective, involving both differential repression and differential activation of specific gene subsets. The overwhelming majority of these genes were not previously known to be regulated by CO_2_. Furthermore, gene ontogeny analysis showed enrichment of hypercapnia-regulated genes involved in a variety of fundamentally important cellular processes. Altering expression of genes related to these processes would be expected to impart functional changes in bronchial epithelial cells, which could in turn influence the pathophysiology and outcomes of many respiratory diseases.

Our data show that hypercapnia alters expression of multiple components of the innate immune system, including downregulation of the IL-6 receptor (*IL6R*); the neutrophil chemokines *CXCL1, CXCL2 and CXCL6*^28^; the mucosal-associated chemokines *CCL28 and CXCL14*^29–31^ and importantly *TLR4*. Hypercapnia also upregulated CD55 and CD86, which bind virus at the cell surface^32,33^. While hypercapnia downregulated TLR4, it increased the expression of *IL1RL1*, which has been shown to inhibit TLR4 activation^34^. Of note, Schneberger *et al*. previously reported that hypercapnia reduced LPS-induced secretion of IL-6 and IL-8 in the airway epithelial cell line BEAS-2B^35^. These observations are relevant because of the well-documented role of TLR4 in host defense against multiple respiratory pathogens^36–40^. Interestingly, airway epithelial TLR4 expression was reduced in patients with severe COPD as compared to those with less severe COPD^41^, possibly due to hypercapnia in patients with advanced disease. Reduced expression of immune response genes was also seen in the lungs of newborn mice exposed to moderate hypercapnia (8% CO_2_) for the first two weeks of life^42^. While the immune genes downregulated by hypercapnia in the newborn mice differed from those we found in NHBE cells, the mucosal immunity chemokine CXCL14^43^ was commonly downregulated in both systems. Taken together, these observations indicate that the airway epithelium is an important target for hypercapnic suppression of innate immune gene expression. This, along with the suppressive effects of elevated CO_2_ on macrophage, neutrophil, alveolar epithelial cell functions^13–15,17–19^ likely contributes to the deleterious impact of elevated CO_2_ on lung injury and host defense.

Another cluster impacted by hypercapnia includes genes related to nucleosome assembly, which also have antibacterial properties. The nucleosome consists of 145–147 base-pair-segments of DNA wrapped around a histone octamer containing one (H3–H4)_2_ tetramer, two H2A–H2B dimers, and histone chaperones or linkers that facilitate nucleosome assembly^44^. Regulation of nucleosome assembly following DNA replication, DNA repair and gene transcription is critical for the maintenance of genome stability and epigenetic information^44^. Within this gene cluster, hypercapnia downregulated transcripts for the core histones H2A and H2B^27^, the histone chaperone NAP1L1^45^, and the linker histone H1^27^. Downregulation of histone gene expression can be triggered by DNA-damage or indirect inhibition of DNA synthesis^46^ and might lead to alterations of chromatin structure that would influence transcriptional regulation of many genes and even genome stability^47^. Exchange of core histones with histone variants might also alter the chemical nature and physical properties of the nucleosome, thereby affecting distinct cellular processes^48^. In addition, histones H2A and H2B also can inactivate endotoxin and function as antimicrobial proteins^49,50^.

We also found that elevated CO_2_ upregulated NHBE cell expression of cholesterol and fatty acid biosynthesis genes, while downregulating ATP-binding cassette (ABC) transporters, which promote the efflux of cholesterol and phospholipids from the cell^51^. Interestingly, enveloped viruses subvert preexisting lipids for viral entry and trafficking and also reprogram lipid synthesis and lipid distribution in lipid rafts to establish an optimal environment for their replication, assembly and egress^52^. Furthermore, host defense against viral infection involves interferon-mediated downregulation of sterol biosynthesis^53^. Thus, hypercapnia-induced cholesterol accumulation might contribute to the entry, replication, and shedding of respiratory viruses in the airways.

As noted above, in a previous study, we showed that hypercapnia downregulates the TCA cycle enzyme IDH2, resulting in mitochondrial dysfunction and impaired proliferation of fibroblasts and A549 lung epithelial cells^20^. However, in the current study, hypercapnia did not alter IDH2 expression in NHBE cells, indicating that CO_2_-mediated regulation of gene expression is cell-type-specific. On the other hand, a number of genes involved in mitochondrial function were regulated by hypercapnia in NHBE cells. Among these, upregulated genes included acyl-CoA dehydrogenase short/branched chain (*ACADSB*) and acyl-CoA synthetase short chain family member 2 (*ACSS2*), which encode enzymes involved in fatty acid synthesis and oxidation^54^. Genes downregulated by elevated CO_2_ included gamma-butyretaine hydroxlase 1 (*BBOX 1*), which catalyzes synthesis of L-carnitine, an essential co-factor in beta-oxidation^55^; kynurenine 3-monooxygenase (*KMO*), an outer mitochondrial membrane protein that hydroxylates tryptophan to form kynurenine^56^; BCL2 interacting protein 3 (*BNIP3*), a BH3 domain protein with pro-apoptotic activity^57^; and mitochondrial assembly of ribosomal large subunit 1 (*MALSU1*), an inhibitor of translation at the mitochondrial ribosome^58^. The diverse activities of these genes indicate the potential for hypercapnia to disrupt multiple mitochondrial functions in NHBE cells.

While the current study does not reveal the molecular mechanism(s) underlying hypercapnia-induced changes in gene transcription, other recent work suggests a path to elucidating components of a putative CO_2_-induced signaling pathway leading to inhibition of innate immune gene expression and impaired host defense. We previously reported that elevated CO_2_ inhibits expression of antimicrobial peptide genes and suppresses antibacterial host defense in *Drosophila*^59^, suggesting that the immunosuppressive effect of hypercapnia is evolutionarily conserved. Using a genome-wide RNAi screen, we identified a small number of genes whose expression is required for CO_2_-induced immunosuppression in *Drosophila* cells, and which are conserved in mammalian systems^60^. Flies deficient in of one these genes, a zinc finger homeodomain transcription factor known as *zfh2*, were protected from CO_2_-induced mortality associated with bacterial infection^60^. This opens up the opportunity to test whether orthologues of *zfh2* and other genes identified in the *Drosophila* screen mediate hypercapnic immunosuppression in mice and ultimately in humans.

Alterations in expression of innate immune and other genes in airway epithelial cells may be of central importance in the CO_2_-induced increase in mortality of Pseudomonas pneumonia we previously observed in mice^17^. In addition, the suppressive effect of elevated CO_2_ on immune gene expression in the airway epithelium, along with similar effects on immune cells, suggest a reason why severe COPD and other lung disease associated with hypercapnia all carry a high risk of pulmonary infection. Bacterial and viral infections are a principal cause of acute COPD exacerbations^61–64^, which are linked to the need for hospitalization and to mortality^65,66^. Thus, CO_2_-induced alterations in airway epithelial gene expression may underlie the increase in mortality associated with hypercapnia in advanced COPD, as well as community-acquired pneumonia^9^, adenoviral lung infections^10^ and cystic fibrosis^11^. It is notable in this regard that reducing hypercapnia with noninvasive ventilatory support has been shown to decrease hospital readmissions and mortality in patients with severe COPD^67,68^. Further investigation of the molecular mechanisms and mediators of CO_2_ effects on gene expression may reveal targets for pharmacologic intervention to prevent hypercapnic immune suppression in patients with advanced respiratory disease.

## Methods

### Primary Normal Human Bronchial Epithelial Cells

Primary NHBE cells isolated from airways of humans without known lung disease were obtained from a commercial source (Lonza). The cells were plated on collagen-coated plastic dishes, grown to confluence in BEGM^TM^ Bronchial Epithelial Cell Growth Medium (Lonza), and passaged after enzyme dissociation with trypsin^69^. Cells from passage-3 were seeded onto 24-mm, 0.4 μm pore size, polyester, transwell inserts (Corning) at 0.5 × 10^6^ cells per insert (4.67 cm^2^) and cultured in a serum-free medium^70^, comprised of 1:1 mixture of BEBM (Lonza): DMEM (Mediatech), supplemented with hydrocortisone (0.5 μg/ml), insulin (5 μg/ml), transferrin (10 μg/ml), epinephrine (0.5 μg/ml), triiodothyronine (6.5 ng/ml), epidermal growth factor (0.5 ng/ml), retinoic acid (50 nM), bovine pituitary extract (0.4%), gentamycin (50 μg/ml), and amphotericin B (50 ng/ml). After the cells reached confluence in submersion culture, the medium above the inserts was removed and the cells were maintained in ALI culture for two more weeks, at which point differentiation to a pseudostratified mucociliary epithelium with characteristics of airway epithelium *in vivo* was established^69,71^. Differentiation after ∼2 wk on ALI culture was confirmed by the presence of beating cilia and mucus production, as previously described^72^. Culture of NHBE cells up to the point of full differentiation was carried out in an atmosphere of humidified 5% CO_2_/95% air at 37 °C.

### Hypercapnia Exposure

After differentiation, NHBE cells were cultured in ALI for an additional 24 h in humidified 20% CO_2_/21% O_2_/59% N_2_ (hypercapnia) or maintained in humidified 5% CO_2_/95% air (5% CO_2_/20% O_2_/75% N_2_; normocapnia), as control. The growth medium was pre-saturated with appropriate CO_2_ concentration for 4 h prior to the addition to the cells. The PCO_2_ and pH of the pre-saturated media were measured using a pHOx Plus Blood Gas Analyzer (Nova Biomedical Corp). For the normocapnia- and hypercapnia-equilibrated media, the PCO_2_s were 44 and 112 mmHg, and the corresponding pH values were 7.4 and 7.1 respectively.

### Cytotoxicity Assay

To determine whether hypercapnia induces cytotoxicity, lactate dehydrogenase (LDH) release to the apical and basolateral compartments was assessed using a colorimetric Cytotoxicity Detection Kit (Roche) according to the manufacturer’s instructions. Absorbance at 490 nm was measured using a VersaMax Tunable Microplate Reader (Molecular Devices). Percent LDH release was calculated as the amount of LDH measured in the basolateral supernatant or apical wash divided by the total amount of LDH in the culture (LDH in cell lysates plus that measured in apical and basolateral compartments) times 100.

### RNA Isolation and Affymetrix GeneChip Hybridization

Total RNA was isolated using the RNeasy Mini kit (Qiagen). Quality and quantity of each RNA sample were assessed using a 2100 BioAnalyzer (Agilent). RNA was hybridized to GeneChip® Human Genome U133 2.0 Plus Array (Affymetrix). A total of 6 chips, each hybridized to a cRNA from different normocapnic (n = 3) or hypercapnic (n = 3) NHBE cell cultures were used in this study. The U133 2.0 Plus Arrays contain probes for approximately 56,921 transcripts and variants, including over 45,000 well characterized human genes. Fluorescent images were detected in a GeneChip® Scanner 3000 and expression data were extracted using the GeneChip Operating System v 1.2 (Affymetrix).

### Microarray Data Analysis

Differential gene expression between normocapnia and hypercapnia was assessed by a statistical linear model analysis using the BioConductor package limma^73,74^ (https://www.bioconductor.org/help/faq/), in which an empirical Bayes method is used to moderate the standard errors of the estimated log-fold changes of gene expression. The moderated t-statistic p-values derived from the limma analysis were further adjusted for multiple testing by Benjamini and Hochberg’s method^75^ to control false discovery rate (FDR). Many genes whose expression signals were below background were defined as “absent”. Transcripts absent in all samples were filtered out, leaving 54,675 probes corresponding to 20,390 genes in the downstream analysis. The lists of differentially expressed genes were obtained by the FDR criteria of <0.05 and fold-change cutoff of >1.4. Differential gene expression in hypercapnia versus normocapnia was depicted in a pie chart, volcano plot of statistical significance (−log_10_ P value) plotted against log_2_ fold change, and hierarchical clustering by Pearson correlation represented as heat maps generated using Heatmapper^76^ and Gene-E (https://software.broadinstitute.org/GENE-E/).

Over representation analysis (ORA) of gene ontology (GO) terms from biological processes of all genes downregulated or upregulated by hypercapnia were separately analyzed using the Gene Ontology Analysis InnateDB tool^77^ which utilizes a manually-curated knowledgebase of genes, proteins, interactions and signaling pathways involved in mammalian innate immune responses. Results from the Innate DB analysis were confirmed using GeneGo Metacore (Thomson Reuter), a separately curated database and pathway analysis tool. Microarray data have been deposited to the National Center for Biotechnology Information (NCBI) Gene Expression Omnibus (GEO; http://www.ncbi.nlm.nih.gov/projects/geo) complied with MIAME standards (accession number GSE110362).

### Network Ontology Analysis

Subsequent analysis of global expression changes and ontology network assessment on the differentially selected genes was performed using Mathematica® v11.2 (Wolfram Research, Inc., Mathematica, Version 11.2, Champaign, IL (2017)). Ontology groups were generated using inbuilt GenomeData, matching the annotated genes with pre-defined processes and intracellular functions. Two approaches were used for analysis of genome wide expression changes: unbiased measurements of intra-network gene expression and fold-change ranked segmentation. Unbiased intra-network changes were assessed for cellular processes that contained at least five genes in the post-screen data. Mean-fold change, the variance of the fold-change, and Pearson correlation of expression were measured for each process. Intra-network heterogeneity of relative expression was measured by calculating the standard deviation of the relative expression for genes within any given ontological process. For instance, if a gene was classified as belonging to both “Nucleosome Assembly” and “Signal Transduction”, it was assigned to both groups and a connection between these processes was indicated. To further understand the impact of hypercapnia-induced differential gene expression, cluster domains of GO biological processes containing 5 or more genes and with at least 4 connections were also generated using Mathematica® v11.2. These processes were broadly grouped based on gene function and by their connections.

### Quantitative TaqMan Real-time RT-PCR

Total RNA was isolated from NHBE cells and first-strand cDNA was generated using MultiScribe™ MuLV reverse transcriptase (Applied Biosystems). The first-strand cDNA was used to quantitate the mRNA levels by TaqMan real-time PCR system (Applied Biosystems). The level of expression of eukaryotic translation elongation factor 1 alpha 1 (*EEF1A1*) was used as reference, and fold change of target genes was calculated by the ∆∆_CT_ method^78^.

### Immunofluorescence Staining for TLR4

After exposure to normocapnia (5% CO_2_) or hypercapnia (20% CO_2_) for 24 h, differentiated NHBE cells were fixed with ice-cold 50% acetone/50% methanol for 5 min. Cells were blocked in PBS containing 2% BSA and 0.1% triton X-100 then double-stained with 1:200 polyclonal rabbit anti-human TLR4 antibody (H-80, Santa Cruz Biotechnology) followed by 1:200 Alexa Fluor 555-conjugated goat-anti-rabbit IgG (red) (Invitrogen), and 1:500 monoclonal mouse anti-human acetylated tubulin antibody (Clone 6–11B-1, Sigma) followed by 1:200 Alexa fluor 488-conjugated goat anti-mouse IgG (green) (Invitrogen). Nuclei were identified by staining with 1 µg/ml Hoescht (blue) (Sigma). Images were obtained using a Nikon TE200 inverted fluorescence microscope (Nikon) equipped with a SPOT RT Monochrome Digital Camera (Diagnostic Instruments). All images were captured with the same gain and exposure time using Metamorph software.

### Immunoblotting for TLR4

After exposure to normocapnia (5% CO_2_) or hypercapnia (20% CO_2_) for 24 h, differentiated NHBE cells were lysed in RIPA buffer (Santa Cruz Biotechnology) supplemented with PMSF, sodium orthovanadate and protease inhibitor cocktail. Lysate proteins (30 μg/well) were resolved by SDS/PAGE 4–20% gradient gels and transferred to nitrocellulose (Bio-Rad Laboratories). Membranes were probed with polyclonal rabbit anti-human TLR4 (H-80) antibody followed by HRP-conjugated anti-rabbit secondary antibody (Pierce). Blots were stripped and re-probed with monoclonal mouse anti-human β-actin (Abcam) followed by HRP-conjugated anti-mouse secondary antibody (Pierce) to confirm the equal loading. The signals were detected using enhanced chemiluminescence SuperSignal West Dura Substrate kit (Pierce). TLR4/βactin ratios were assessed using ImageJ^79^.

### Statistical analysis

Data are presented as means ± SE. Differences between two groups were assessed using Student’s t test. Levene’s test was used to analyze the homogeneity of variances. Significance was accepted at p < 0.05.

## Electronic supplementary material

Supplementary Information
