# Supplementary material for: Hypercapnia Alters Expression of Immune Response, Nucleosome Assembly and Lipid Metabolism Genes in Differentiated Human Bronchial Epithelial Cells
Source: Sci Rep. 2018 Sep 10;8:13508. doi: 10.1038/s41598-018-32008-x (PMC6131151; doi:10.1038/s41598-018-32008-x)
Supplement: Supplementary file 1 — Supplementary Information [file 41598_2018_32008_MOESM1_ESM.docx]

**Hypercapnia Alters Expression of Immune Response, Nucleosome Assembly and Lipid Metabolism Genes in Differentiated Human Bronchial Epithelial Cells**

S. Marina Casalino-Matsuda **^1*#^**, Naizhen Wang **^1#^**, Peder Thusgaard Ruhoff **^2^**, Hiroaki Matsuda **^3^**, Marie C. Nlend ^4^, Aisha Nair **^1^**, Igal Szleifer **^5,6,7^**, Greg J. Beitel **^8^**, Jacob Iasha Sznajder **^1^**, Peter H. S. Sporn **^1,9^**

Supplementary information


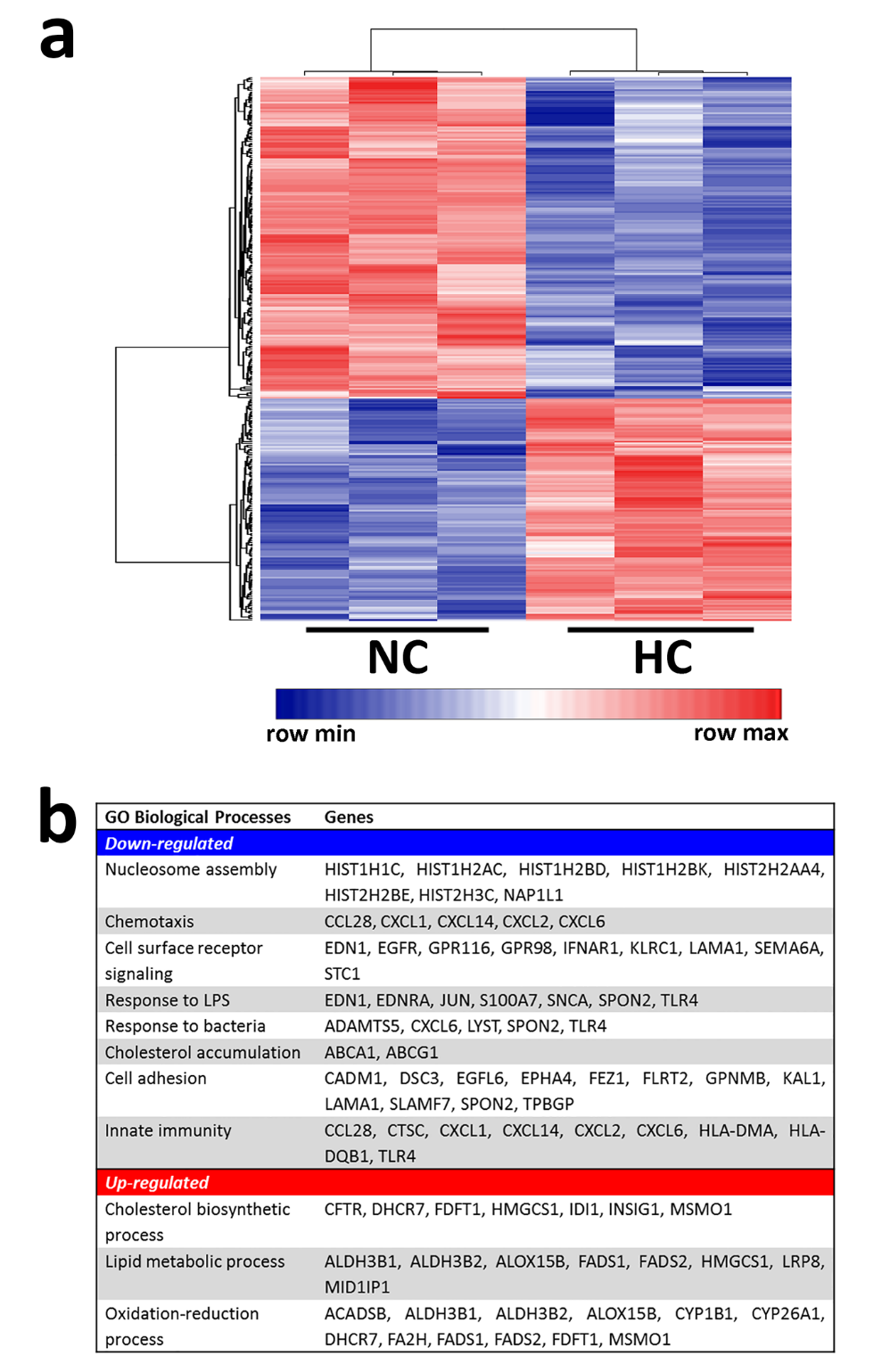


**Supplementary Figure 1: Hypercapnia induces transcriptional changes in NHBE cells.**

**a)** Heat map with hierarchical clustering of gene expression profiles in NHBE cells in hypercapnia vs normocapnia. Each column represents one sample and each row represents one transcript. **b)** Selected genes related to major CO_2_-regulated GO biological processes.


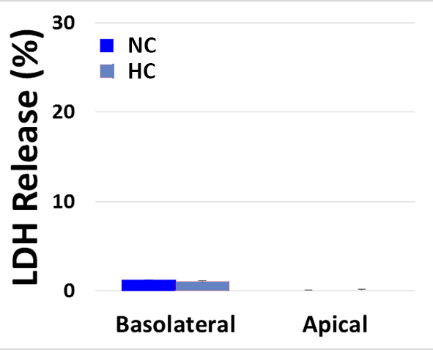


**Supplementary Figure 2: Hypercapnia is not cytotoxic for NHBE Cells.** Differentiated NHBE cells were exposed to 5% CO_2_ (normocapnia, NC), or 20% CO_2_ (hypercapnia, HC) for 24 h. Cytotoxicity was assessed by determination of LDH release in the basolateral medium (lower chamber) and apical wash (upper chamber) as a percentage of the total LDH in the culture. Results shown are means ± SE; n = 3.


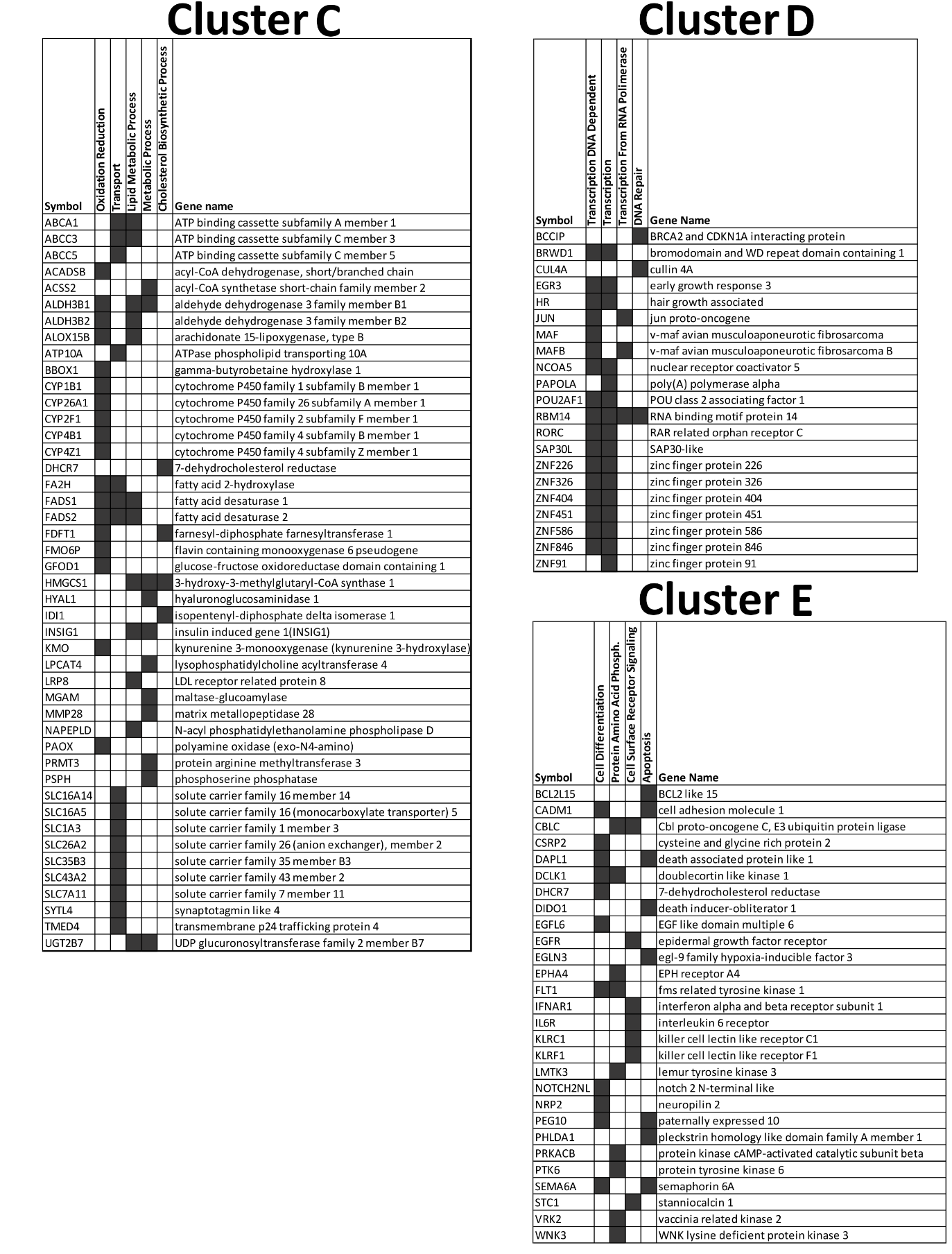


**Supplementary Figure 3:** List of genes altered by hypercapnia and their associated GO biological processes in clusters C, D, and E.


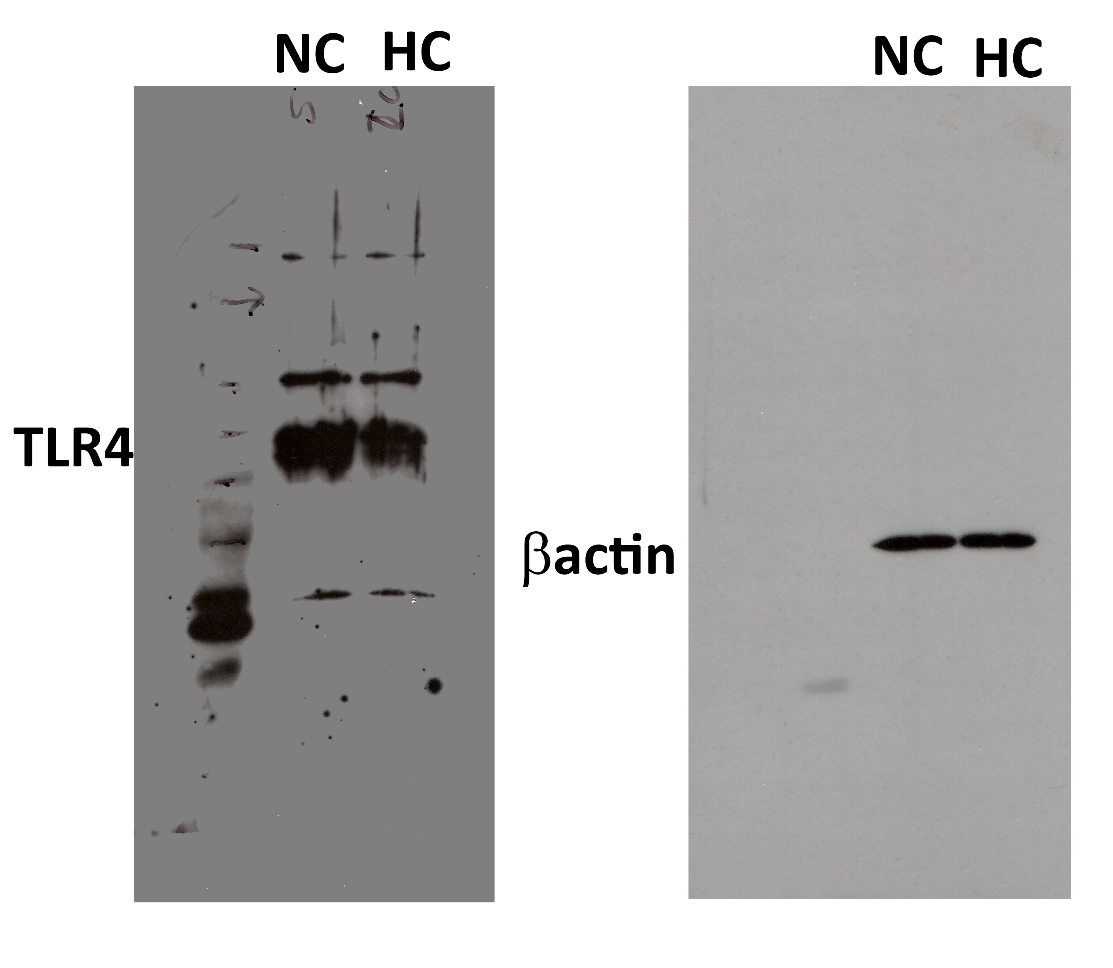


**b**

**a**

**Supplementary Figure 4: Hypercapnia alters expression of TLR4.** ALI-differentiated NHBE cells were exposed to normocapnia (NC) or hypercapnia (HC) for 24 h prior to analysis. Representative immunoblot of whole cell lysates for TLR4 (**a**) and β-actin (**b**).

**Supplementary Table 1: Genes Downregulated in NHBE Cells Following Exposure to Hypercapnia (20% CO_2_) for 24 h**

**Supplementary Table 1: Genes Downregulated in NHBE Cells Following Exposure to Hypercapnia (20% CO_2_) for 24 h (continuation)**

**Supplementary Table 1: Genes Downregulated in NHBE Cells Following Exposure to Hypercapnia (20% CO_2_) for 24 h (continuation)**

**Supplementary Table 1: Genes Downregulated in NHBE Cells Following Exposure to Hypercapnia (20% CO_2_) for 24 h (continuation)**

**Supplementary Table 2: Genes Upregulated in NHBE Cells Following Exposure to Hypercapnia (20% CO_2_) for 24 h**

**Supplementary Table 2: Genes Upregulated in NHBE Cells Following Exposure to Hypercapnia (20% CO_2_) for 24 h (continuation)**

**Supplementary Table 2: Genes Upregulated in NHBE Cells Following Exposure to Hypercapnia (20% CO_2_) for 24 h (continuation)**
